# PKC regulates the production of fibroblast growth factor 23 (FGF23)

**DOI:** 10.1371/journal.pone.0211309

**Published:** 2019-03-28

**Authors:** Ludmilla Bär, Philipp Hase, Michael Föller

**Affiliations:** 1 Institute of Agricultural and Nutritional Sciences, Martin Luther University Halle-Wittenberg, Halle (Saale), Germany; 2 Institute of Physiology, University of Hohenheim, Stuttgart, Germany; China Medical University, TAIWAN

## Abstract

Serine/threonine protein kinase C (PKC) is activated by diacylglycerol that is released from membrane lipids by phospholipase C in response to activation of G protein-coupled receptors or receptor tyrosine kinases. PKC isoforms are particularly relevant for proliferation and differentiation of cells including osteoblasts. Osteoblasts/osteocytes produce fibroblast growth factor 23 (FGF23), a hormone regulating renal phosphate and vitamin D handling. PKC activates NFκB, a transcription factor complex controlling FGF23 expression. Here, we analyzed the impact of PKC on FGF23 synthesis. *Fgf23* expression was analyzed by qRT-PCR in UMR106 osteoblast-like cells and in IDG-SW3 osteocytes, and FGF23 protein was measured by ELISA. Phorbol ester 12-O-tetradecanoylphorbol-13-acetate (PMA), a PKC activator, up-regulated FGF23 production. In contrast, PKC inhibitors calphostin C, Gö6976, sotrastaurin and ruboxistaurin suppressed FGF23 formation. NFκB inhibitor withaferin A abolished the stimulatory effect of PMA on *Fgf23*. PKC is a powerful regulator of FGF23 synthesis, an effect which is at least partly mediated by NFκB.

## Introduction

Protein kinase C (PKC) isoforms are related serine/threonine kinases probably expressed in all cell types. Classically, PKC activity is induced upon stimulation of various G_q_ protein-coupled receptors and growth factor receptor tyrosine kinases [1]. Three classes of PKC isoforms can be distinguished: classical PKC (cPKC) isoforms are activated by both, diacylglycerol (DAG) and an increase in the intracellular Ca^2+^ concentration whereas novel PKC (nPKC) isoforms require only DAG, and atypical PKC (aPKC) isoforms are induced by other mechanism [2]. The classical activation is dependent on phospholipase Cβ or Cγ-mediated breakdown of membrane phosphatidylinositol 4,5-bisphosphate (PIP_2_) yielding inositol 1,4,5-trisphosphate (IP_3_) and DAG. IP_3_ binds the IP_3_ receptor releasing Ca^2+^ from the endoplasmic reticulum (ER), while membrane-bound DAG activates PKC [1].

PKC is crucial for most cellular responses including the regulation of gene expression, cell migration, proliferation, differentiation, and apoptosis [3]. Moreover, PKC is implicated in the pathophysiology of frequent disorders such as heart failure, diabetes, Alzheimer and Parkinson disease, as well as inflammatory and immune disorders [3]. PKC is particularly relevant for various malignancies, owing to its tumor and metastasis-promoting properties [3]. Plant-derived phorbol esters are potent carcinogens that are effective through stimulating PKC activity [3].

Inflammation [4,5], renal and cardiovascular disease [6–8] are major triggers of the production of fibroblast growth factor 23 (FGF23), a proteohormone mainly produced in the bone [9] and implicated in the regulation of phosphate reabsorption and 1,25(OH)_2_D_3_ (active vitamin D [10]) formation in the kidney [11]. The FGF23-mediated inhibition of renal phosphate transporter NapiIIa and CYP27B1, the key enzyme for the generation of 1,25(OH)_2_D_3_, is dependent on Klotho, a transmembrane protein [9]. The induction of left ventricular hypertrophy is, however, solely mediated by FGF23 without the involvement of Klotho [8,12,13], whereas vitamin D partly overcomes the FGF23 effect on cardiac hypertrophy [14]. FGF23 also impacts on neutrophil recruitment [15], erythropoiesis [16,17], or hepatic cytokine secretion [18] in a paracrine manner.

Klotho or FGF23 deficiency results in rapid aging, a very short life span, and multiple age-associated diseases affecting most organs and tissues [11,19]. This dramatic phenotype of Klotho or FGF23 null mice is almost completely rescued by a phosphate- or vitamin D-deficient diet [20,21], pointing to the predominant role of calcification in the pathophysiology of Klotho or FGF23 deficiency [22]. Apart from its endocrine and paracrine effects that are still incompletely understood, FGF23 is a putative disease biomarker [6,23]. The plasma level of FGF23 correlates well with progression of chronic kidney disease (CKD) and is a very sensitive marker that is elevated even before onset of hyperphosphatemia or hyperparathyroidism [24,25]. Furthermore, FGF23 levels are elevated in acute kidney injury [26,27]. Whether and to which extent FGF23 not only indicates disease but actively contributes to disease progression, as shown for the heart, remains unclear.

The identification of molecular regulators of FGF23 production is of high interest and relevance. Known regulators include PTH [28], 1,25(OH)_2_D_3_ [29], phosphate [30,31], inflammatory cytokines and factors such as tumor necrosis factor α (TNFα) [32,33], interleukin (IL)-1/6 [4,33–35], NFκB [33,36], transforming growth factor (TGF)β [37], AMP-dependent protein kinase (AMPK) [38] or insulin-dependent PI3 kinase signaling [39].

The present study explored the contribution of PKC signaling to the production of FGF23 in bone cells.

## Materials and methods

### Cell culture

Cell culture and experiments with UMR106 rat osteoblast-like cells (purchased from ATCC, Manassas, VA, USA) were conducted as described before [39]. Briefly, cells were cultured in DMEM high-glucose medium containing 10% FBS and penicillin-streptomycin at 37°C and 5% CO_2_.

IDG-SW3 bone cells (purchased from Kerafast, Boston, MA, USA) were cultured as described earlier [40]. Briefly, non-differentiated cells were kept at 33°C in AlphaMEM medium (with L-glutamine and deoxyribonucleosides) containing 10% FBS, penicillin-streptomycin and interferon-gamma (INF-γ; 50 U/ml). For differentiation, cells were plated on collagen-coated dishes at 37°C in medium with 50 μg/ml ascorbic acid and 4 mM β-glycerophosphate but without INF-γ. All reagents were from ThermoFisher unless indicated.

IDG-SW3 osteocytes were used after 35 days of differentiation, and UMR106 cells were pretreated with 100 nM 1,25(OH)_2_D_3_ (Tocris, Wiesbaden-Nordenstadt, Germany) for 24h before the experiment. Cells were then incubated with activator phorbol ester 12-O-tetradecanoylphorbol-13-acetate (PMA; Sigma, Schnelldorf, Germany; 0.1 μM; 6 h) with or without 1 μM PKC inhibitors calphostin C (Tocris), Gö6976 (Tocris), sotrastaurin (Selleckchem, München, Germany), ruboxistaurin (Selleckchem), or NFκB inhibitor withaferin A (Tocris; 0.5 μM), or with vehicle only for another 24 h.

### Expression analysis

Total RNA was extracted using peqGOLD TriFast reagent (VWR, Dresden, Germany). Complementary DNA (cDNA) was synthesized from 1.2 μg RNA using random primers and the GoScript^™^ Reverse Transcription System (Promega, Mannheim, Germany) at 25°C for 5 min, 42°C for 1 h, and 70°C for 15 min.

The expression profile of PKC isoforms in UMR106 cells was studied by RT-PCR using the GoTaq Green Master Mix (Promega) and the primers listed in Table 1. For PCR, 2 μl synthesized cDNA were used. Settings were: 95°C for 3 min, followed by 35 cycles of 95°C for 30 s, 60°C for 30s, 72°C for 45s. PCR products were loaded on a 1.5% agarose-gel and visualized by Midori Green (Biozym, Hessisch Oldendorf, Germany), and a 100 bp DNA ladder (Jena Bioscience, Jena, Germany) was used as a size marker.

**Table 1:** Primer sequences used for expression analysis.

name	sequence (5'-3')	product
*Pkcα*_forward	CTGAACCCTCAGTGGAATGAGT	326 bp
*Pkcα*_reverse	GGCTGCTTCCTGTCTTCTGAA	
*Pkcβ*_forward	AACGGCTTGTCAGATCCCTA	250 bp
*Pkcβ*_reverse	CCACTCCGGCTTTCTGTAGT	
*Pkcγ*_forward	AGTCCCACGGACTCCAAGAG	397 bp
*Pkcγ*_reverse	CGGCATAGAATGCTGCGTG	
*Pkcδ*_forward	TGTGAAGACTGCGGCATGAA	265 bp
*Pkcδ*_reverse	AGGTGAAGTTCTCAAGGCGG	
*Pkcε*_forward	CGAGGACGACTTGTTTGAATCC	389 bp
*Pkcε*_reverse	CAGTTTCTCAGGGCATCAGGTC	
*Pkcζ*_forward	GTGGACCCCACGACAACTT	207 bp
*Pkcζ*_reverse	GATGCTTGGGAAAACGTGGA	
*Pkcη*_forward	CCATGAAGATGCCACAGGGATC	249 bp
*Pkcη*_reverse	TCATCGATCGGAGTTAAAACAGG	
*Pkcθ*_forward	TGCCGACAATGTAATGCAGC	219 bp
*Pkcθ*_reverse	ACACTTGAGACCTTGCCTCG	
*Pkcι*_forward	CTCCTGATCCACGTGTTCCC	321 bp
*Pkcι*_reverse	GGATGACTGGTCCATTGGCA	

### qRT-PCR

Relative expression levels of *Fgf23* and other relevant genes were determined by qRT-PCR using 2 μl synthesized cDNA (for primers see Table 2), and GoTaq qPCR Master Mix (Promega) on a Rotor-Gene Q (Qiagen, Hilden, Germany). PCR conditions were 95°C for 3 min, followed by 35 cycles of 95°C for 10 s, 58°C for 30 s and 72°C for 45 s. After normalization to *Tbp* (TATA box-binding protein) expression, relative quantification of gene expression was carried out based on the double-delta Ct (threshold cycle) method.

**Table 2:** Primer sequences used for qRT-PCR.

name	sequence (5'-3')
*Tbp*_forward	ACTCCTGCCACACCAGCC
*Tbp*_reverse	GGTCAAGTTTACAGCCAAGATTCA
*Fgf23*_forward	TGGCCATGTAGACGGAACAC
*Fgf23*_reverse	GGCCCCTATTATCACTACGGAG
*Il6*_forward	CAGAGTCATTCAGAGCAATAC
*Il6*_reverse	CTTTCAAGATGAGTTGGATGG
*Tnfα*_forward	CTCACACTCAGATCATCTTC
*Tnfα*_reverse	GAGAACCTGGGAGTAGATAAG
*Nfatc1*_forward	GAAGACTGTCTCCACCACCA
*Nfatc1*_reverse	CCGATGTCTGTCTCCCCTTT
*Nfatc3*_forward	TGATGGCCTTGGATCTCAGT
*Nfatc3*_reverse	CCCTCGGCTACCTTCAGTTT
*Nfatc4*_forward	GAAAGAGATGGCTGGCATGG
*Nfatc4*_reverse	CACCTCAATCCTCAGCTCCA
*Hif1α*_forward	GAAAGGATTACTGAGTTGATGG
*Hif1α*_reverse	CAGACATATCCACCTCTTTTTG
*Phex*_forward	ATGGCTGGATAAGCAATAAC
*Phex*_reverse	GCTTTTTCAATCGCTTTCTC
*Dmp1*_forward	ACTGTTATCCTCCTTACGTTC
*Dmp1*_reverse	GGTCTATACTGGCTTCTGTC

### FGF23 ELISA (C-Term)

UMR106 osteoblast-like cell culture supernatant was collected and frozen at -80°C. The medium was concentrated using Sartorius Vivaspin 6 Centrifugal Concentrators (Sartoritus, Göttingen, Germany). The concentration of C-terminal FGF23 was determined by an ELISA (Immutopics, San Clemente, CA, USA) according to the manufacturer’s instructions.

### Statistics

Arithmetic means ± SEM were calculated, and *n* represents the number of independent experiments. Comparisons of two groups were made by unpaired Student’s t test, and for more than two groups, comparisons were calculated via one-way ANOVA, followed by Tukey’s or Dunnett’s multiple comparison tests, using GraphPad Prism. Differences were considered significant if p < 0.05.

## Results

**Fig 1:**
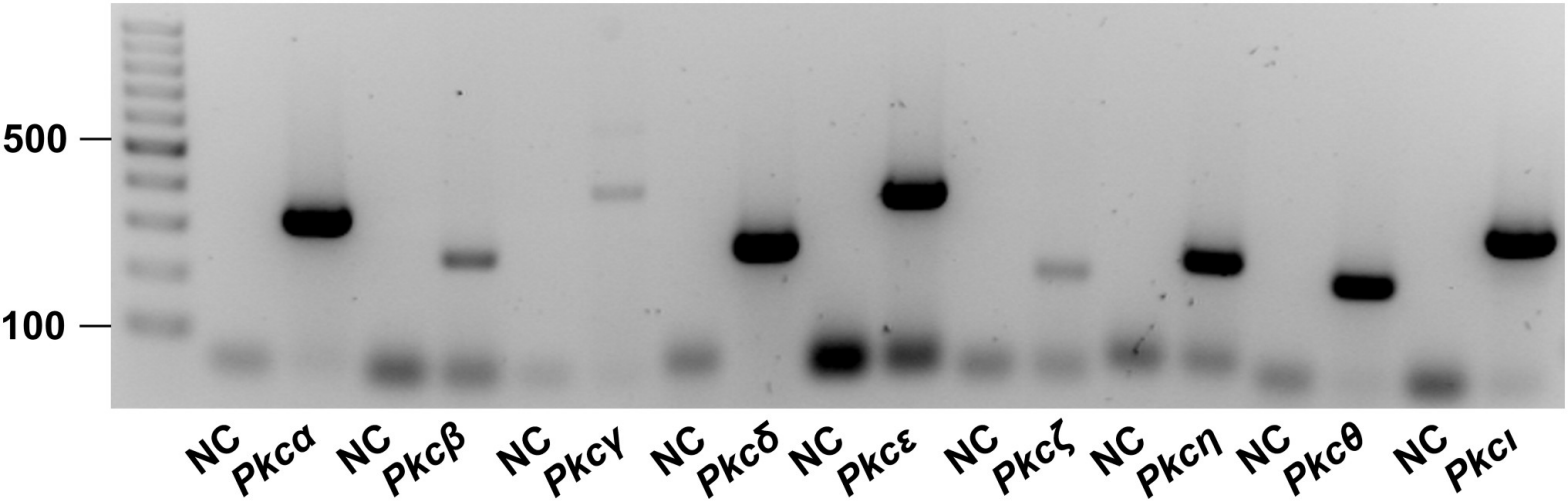
Expression of *Pkc* isoforms in UMR106 osteoblast-like cells. Original agarose gel photo showing *Pkcα*, *-β*, *-γ*, *-δ*, *-ε*, *-ζ*, *-η*, *-θ or -ι* specific cDNA in UMR106 cells. NC: non-template control.

The relevance of PKC activity for the synthesis of FGF23 was studied in UMR106 osteoblast-like cells and IDG-SW3 osteocytes. First, the expression of *Pkc* isoforms was explored by RT-PCR. As demonstrated in Fig 1, mRNA specific for *Pkcα*, *Pkcδ*, *Pkcε*, *Pkcη*, *Pkcθ*, and *Pkcι* could readily be detected. The bands indicating the abundance of *Pkcβ*, *Pkcγ*, *Pkcζ* mRNA in UMR106 cells were weaker albeit detectable.

**Fig 2:**
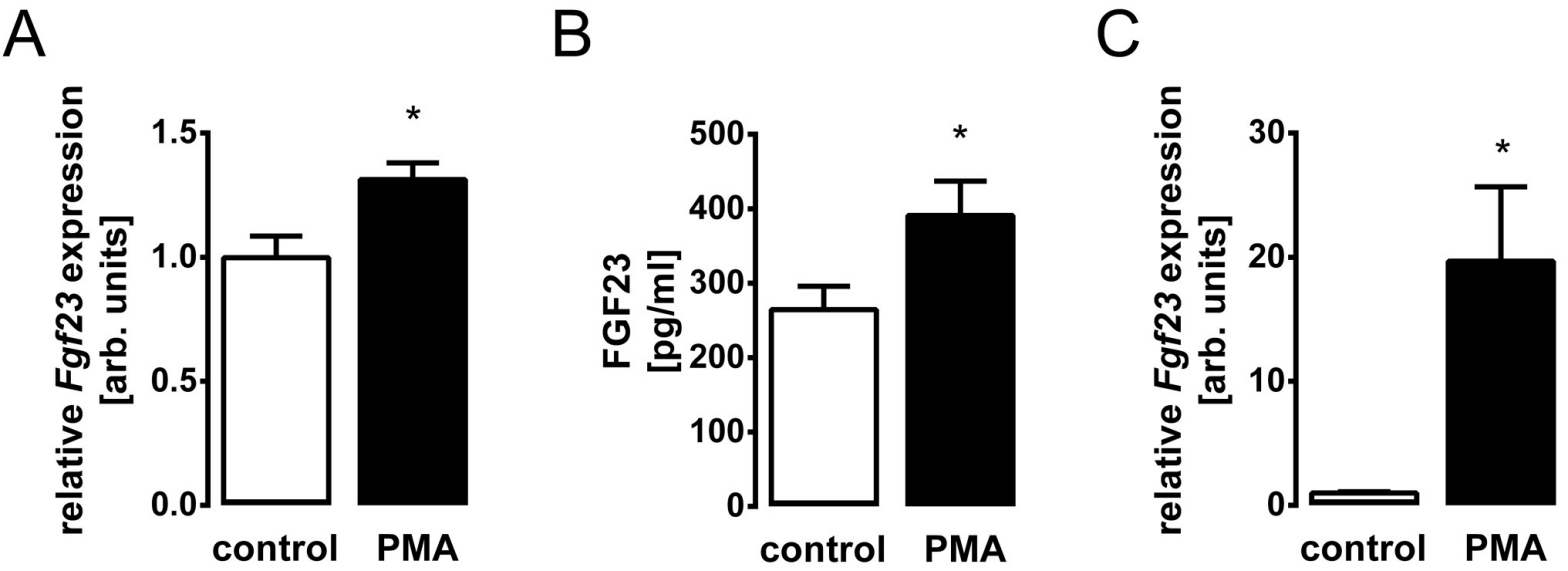
PKC activator PMA induces FGF23 production in UMR106 osteoblast-like cells and in IDG-SW3 osteocytes. Arithmetic means ± SEM (n = 6) of relative *Fgf23* mRNA abundance normalized to *Tbp* in UMR106 osteoblast-like cells (**A**) or IDG-SW3 osteocytes (**C**), and FGF23 concentration in the cell culture supernatant of UMR106 cells (**B**) incubated without (white bars) or with (black bars) 0.1 μM PKC activator PMA. * *p* < 0.05 indicates significant difference. arb., arbitrary.

Phorbol ester 12-O-tetradecanoylphorbol-13-acetate (PMA) is a potent activator of PKC [3]. We treated UMR106 cells with and without PMA and determined *Fgf23* transcripts by qRT-PCR. PMA treatment significantly up-regulated the abundance of *Fgf23* mRNA (Fig 2A). As a next step, we explored whether PMA-stimulated *Fgf23* gene expression translates into enhanced FGF23 production. To this end, we determined FGF23 protein in the supernatant of UMR106 cells. As shown in Fig 2B, PMA indeed stimulated FGF23 synthesis. Similar to osteoblasts, PKC activation with PMA enhanced *Fgf23* gene expression in IDG-SW3 osteocytes (Fig 2C). These results suggest that PKC activity drives *Fgf23* gene expression in osteoblasts and osteocytes.

**Fig 3:**
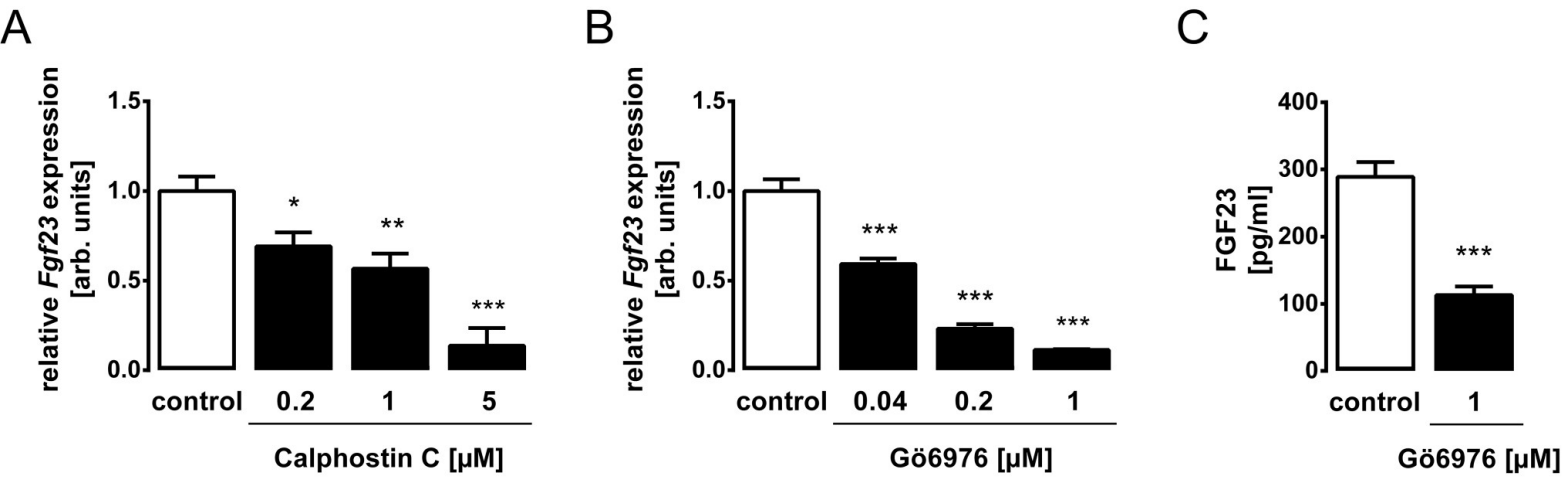
PKC inhibitors Calphostin C and Gö6976 decrease FGF23 expression levels in UMR106 osteoblast cells. UMR106 cells were treated without and with PKC inhibitors Calphostin C (**A**) or Gö6976 (**B, C**) at the indicated concentrations. Arithmetic means ± SEM (n = 6) of the relative *Fgf23* mRNA abundance in UMR106 cells (**A, B**). Gene expression was normalized to *Tbp* as housekeeping gene. Arithmetic means ± SEM (n = 6) of FGF23 protein concentration in the cell culture supernatant (**C**). *p < 0.05, **p < 0.01, and ***p < 0.001 indicate significant difference. arb., arbitrary.

Our next series of experiments tested whether inhibition of PKC interferes with FGF23 expression. To this end, UMR106 cells were exposed to PKC inhibitors. As demonstrated in Fig 3, PKC inhibitor calphostin C (Fig 3A) and also PKCα/β inhibitor Gö6976 (Fig 3B) significantly and dose-dependently down-regulated *Fgf23* gene expression in UMR106 cells. PKCα/β inhibitor Gö6976 also lowered the FGF23 protein concentration in the cell culture supernatant (Fig 3C). Thus, PKC is a stimulator of FGF23 expression.

**Fig 4:**
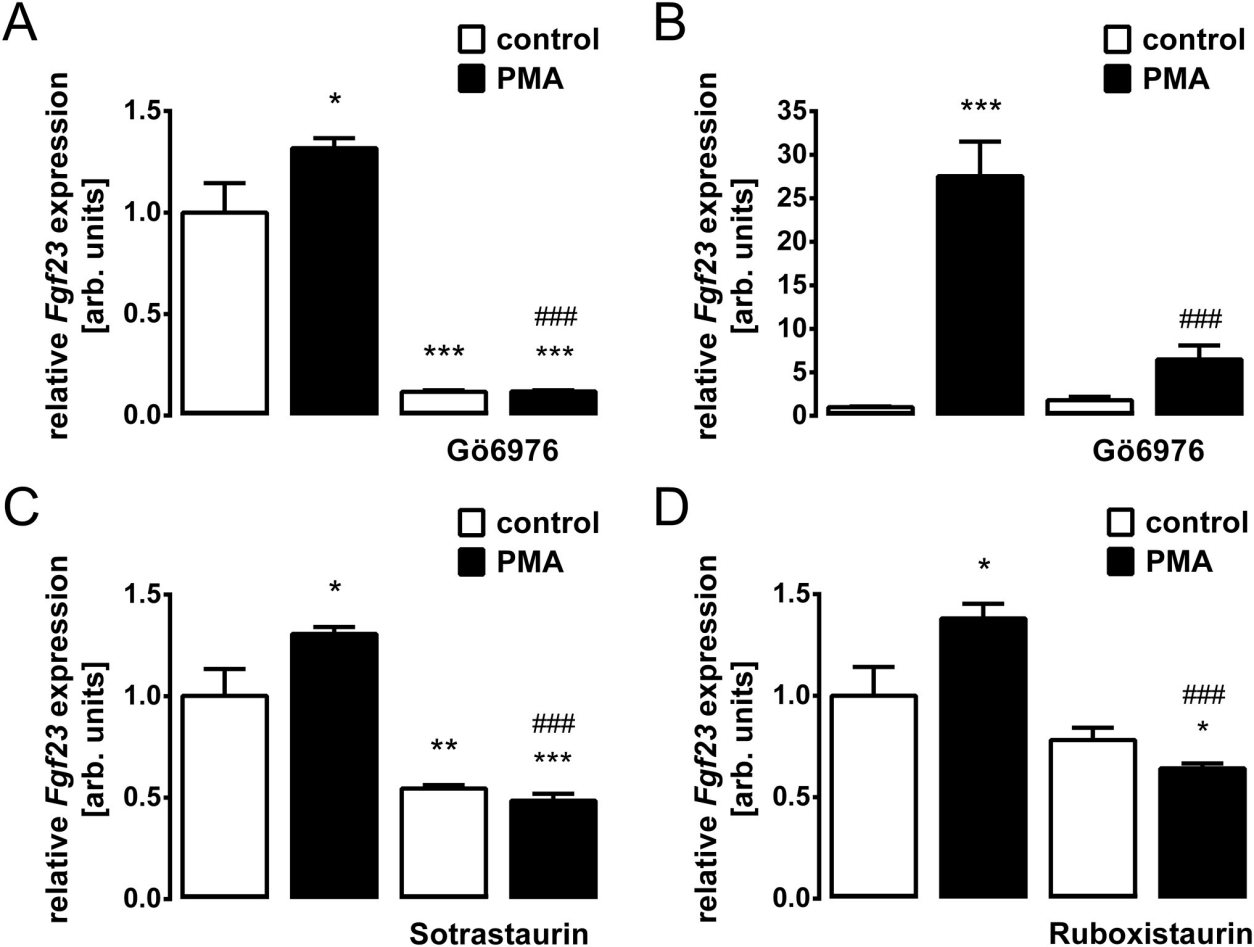
PKC inhibition abrogates the PMA-induced increase in *Fgf23* gene expression in UMR106 osteoblast-like cells and in IDG-SW3 osteocytes. Relative *Fgf23* transcript levels in UMR106 cells (**A,C,D**) or in IDG-SW3 cells (**B**) incubated without or with PMA (0.1 μM, **A-D**) in the absence and presence of PKCα/β inhibitor Gö6976 (1 μM, **A,B**), pan PKC inhibitor Sotrastaurin (1 μM, **C**) or PKCβ inhibitor Ruboxistaurin (1 μM, **D**). Gene expression was normalized to *Tbp* as a housekeeping gene, and the values are expressed as arithmetic means ± SEM (n = 6). *p < 0.05, **p < 0.01, and ***p < 0.001 indicate significant difference from vehicle (first bar). ###*p* < 0.001 indicates significant difference from the absence of PKC inhibitor (second bar vs. fourth bar). arb., arbitrary.

We investigated whether PMA-stimulated *Fgf23* gene expression is indeed dependent on PKC activity using UMR106 and IDG-SW3 cells. As demonstrated in Fig 4, the PMA effect on *Fgf23* gene expression was completely abrogated by PKC inhibitor Gö6976 in UMR106 osteoblast-like cells (Fig 4A) and in IDG-SWR3 osteocytes (Fig 4B), and also by PKC inhibitors sotrastaurin (Fig 4C) and ruboxistaurin (Fig 4D) in UMR106 cells.

**Fig 5:**
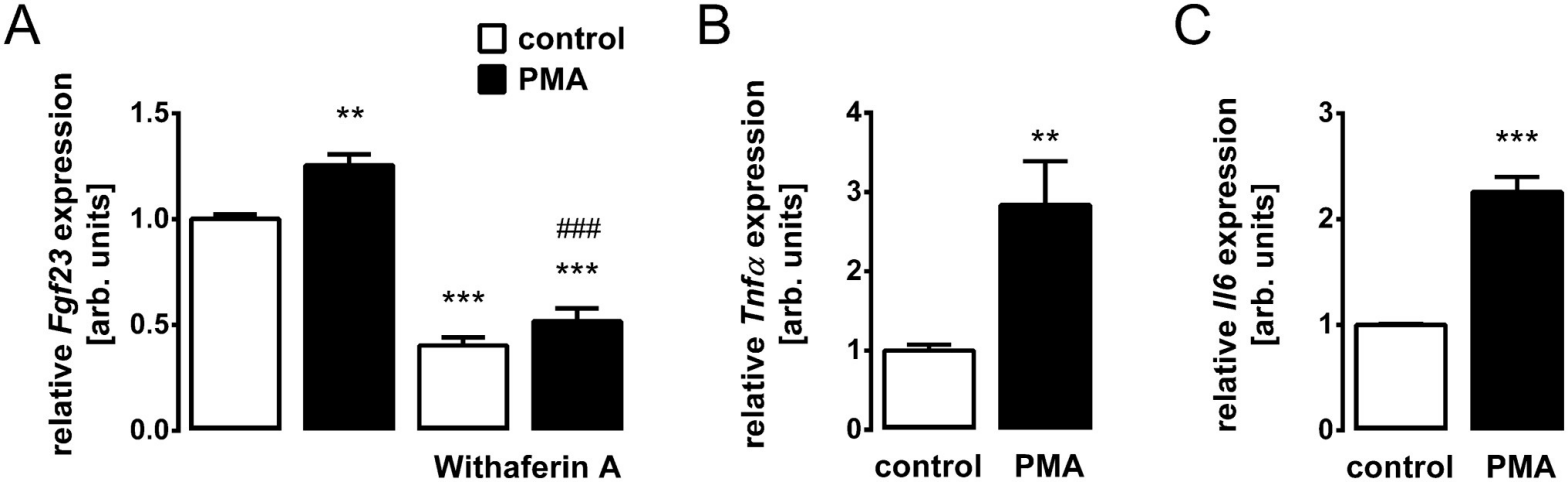
The PKC effect on *Fgf23* gene expression is dependent on inflammation. Relative *Fgf23* transcript levels in UMR106 cells incubated without or with PMA (0.1 μM) in the absence and presence of NFκB inhibitor Withaferin A (0.5 μM) (**A**). Relative *Tnfα* (**B**) and *Il6* (**C**) transcript levels in UMR106 cells incubated without or with PMA (0.1 μM). Gene expression was normalized to *Tbp* as a housekeeping gene, and the values are expressed as arithmetic means ± SEM (n = 6). **p < 0.01, and ***p < 0.001 indicate significant difference from vehicle (first bar). ###*p* < 0.001 indicates significant difference from the absence of withaferin A (second bar vs. fourth bar). arb., arbitrary.

Finally, we sought to identify the mechanism of PKC-dependent FGF23 regulation. Since PKC is an activator of NFκB [41], a known regulator of FGF23, we treated UMR106 cells with and without NFκB inhibitor withaferin A in the absence and presence of PMA and determined *Fgf23* gene expression. As demonstrated in Fig 5A, the PMA effect was indeed abrogated by withaferin A. Hence, PKC was, at least in part, effective through NFκB activity. The pro-inflammatory cytokines TNFα [32,33] and IL-6 [33,35] are also regulators of FGF23 synthesis. We therefore checked whether PKC activity impacts on their expression. According to Fig 5B and 5C PKC activation resulted in enhanced gene expression of *Tnfα* and *Il-6* in UMR106 cells.

Further transcription factors involved in FGF23 production include NFAT [42,43] and HIF-1α [44–46]. Therefore, we tested whether PKC induces the expression of these transcription factors. As a result, incubation of UMR106 cells with 0.1 μM PMA did not significantly affect *Nfatc1* mRNA (control: 1.0 ± 0.02; PMA: 1.03 ± 0.02; n = 6), *Nfatc4* mRNA (control: 1.0 ± 0.03; PMA: 1.02 ± 0.06; n = 6) or *HIF-1α* mRNA (control: 1.0 ± 0.02; PMA: 1.0 ± 0.05; n = 6). The abundance of *Nfat3* mRNA was significantly down-regulated upon incubation with PMA (control: 1.0 ± 0.02; PMA: 0.79 ± 0.03; *p* < 0.001; n = 6).

The peptidase PHEX (phosphate regulating gene with homologies to endopeptidases on the X chromosome) and matrix protein DMP1 (dentin matrix protein-1) are further important regulators of FGF23 [47,48]. PKC activation did not affect *Phex* mRNA (control: 1.0 ± 0.02; PMA: 1.14 ± 0.04; n = 6) but significantly up-regulated *Dmp1* mRNA (control: 1.0 ± 0.02; PMA: 255.76 ± 12.82; *p* < 0.001; n = 6).

## Discussion

Our study discloses PKC as a novel regulator of FGF23 production. We provide experimental evidence that activation of PKC induces and inhibition of PKC suppresses *Fgf23* gene expression: Four different PKC inhibitors were similarly capable of completely blocking PMA-induced *Fgf23* expression. Moreover, altered *Fgf23* gene expression translated into protein secretion as demonstrated by FGF23 protein measurements in the cell culture supernatant. These results unequivocally demonstrate the powerful role of PKC in regulating FGF23 production.

FGF23 is mainly produced by osteoblasts/osteocytes in the bone [9]. The differentiation of osteoblasts is driven by transcription factor MSX2 [49]. PKC enhances the proliferation of osteoblasts [50]. In contrast, PKC inhibits the differentiation of osteoblasts by targeting MSX2 [51] whereas FGF23 induces MSX2. [52]. Transcriptional activity of Runx2, also implicated in osteoblast differentiation, is enhanced by both PKC [53] and FGF23 [54].

We could show that four different pharmacological PKC inhibitors potently suppressed *Fgf23* gene expression. Enhanced PKC activity contributes to the pathophysiology of several disorders, and pharmacological PKC inhibition has therefore been suggested as a therapeutic approach in multiple diseases including cancer, sequelae of diabetes, cardiovascular diseases, or inflammatory disorders such as psoriasis [55]. Some of these diseases, particularly renal and cardiovascular diseases, are associated with elevated FGF23 plasma levels [6], and FGF23 not only indicates disease, but actively contributes at least to left heart hypertrophy [12,13]. It therefore appears to be possible that the therapeutic benefit of PKC inhibition in some of these diseases is at least in part also due its FGF23-lowering properties.

Two different cell lines representing both osteoblasts and mature osteocytes were used in our study to decipher the effect of PKC on FGF23: PKC activation with PMA enhanced and PKC inhibition with different PKC inhibitors suppressed *Fgf23* gene expression in both UMR106 osteoblast-like cells and IDG-SW3 osteocytes. This result suggests PKC-dependent regulation of FGF23 formation as a universal mechanism for the control of this hormone.

In an attempt to identify the underlying mechanism, we found that PKC-mediated up-regulation of *Fgf*23 expression is sensitive to NFκB inhibition. NFκB, as a p65/p50 dimer, is inactivated by binding inhibitory κB (IκB). The inhibitory κB kinase (IKK) phosphorylated IκB, leading to its ubiquitination and degradation, paralleled by the release and activation of NFκB [56]. Therefore, the activation of the IKK complex plays a crucial role in the induction of NFκB, and PKC activates NFκB through this kinase [41]. Clearly, inflammation is a major trigger of FGF23 production [4,36]. In detail, NFκB up-regulates Ca^2+^ release-activated Ca^2+^ (CRAC) channel Orai1/STIM1 accomplishing store-operated Ca^2+^ entry (SOCE) and inducing the transcription of the *Fgf*23 gene [36]. According to our experiment with NFκB inhibitor withaferin A, the PKC effect on FGF23 was, at least partly, dependent on NFκB pointing to the decisive role of this pro-inflammatory transcription factor complex in the regulation of FGF23. Moreover, PKC activation induced the expression of pro-inflammatory cytokines TNFα and IL-6 which are themselves stimulators of FGF23 production [33]. Therefore, the enhanced formation of these cytokines may contribute to the PKC effect on FGF23. NFAT isoforms and HIF1α, further regulators of FGF23, were not affected by PKC activity in UMR106 cells.

We observed a PKC-mediated up-regulation of FGF23 regulator DMP1 in UMR106 cells which would be expected to lower FGF23 levels. Possibly, the PKC effect on FGF23 involving NFκB overcomes the expected down-regulation of FGF23 due to PKC-mediated DMP1 induction. Alternatively, enhanced PKC-stimulated FGF23 formation is limited by counter regulatory DMP1 induction. Clearly, further studies are warranted to address this question. In addition, since our study was carried out *in vitro* only, its *in vivo* relevance will also have to be verified separately.

In conclusion, our study identified PKC as a novel regulator of FGF23 production in both osteoblast and osteocytes, being at least partially effective via NFκB signaling. Therefore, the therapeutic modulation of PKC activity in chronic disease may impact on the plasma FGF23 concentration.
